# Development and validation of a Medication Adherence Universal Questionnaire: the MAUQ

**DOI:** 10.1007/s11096-023-01612-x

**Published:** 2023-06-17

**Authors:** Ana C. Cabral, Marta Lavrador, Margarida Castel-Branco, Isabel Vitória Figueiredo, Fernando Fernandez-Llimos

**Affiliations:** 1 Pharmacology and Pharmaceutical Care Laboratory, Faculty of Pharmacy, University of Coimbra, Coimbra, Portugal; 2 Institute for Clinical and Biomedical Research (iCBR), Faculty of Medicine, University of Coimbra, Coimbra, Portugal; 3 Laboratory of Pharmacology, Department of Drug Sciences, Faculty of Pharmacy, University of Porto, Porto, Portugal; 4 Applied Molecular Biosciences (UCIBIO), University of Porto, Porto, Portugal

**Keywords:** Medication adherence, Psychometrics, Questionnaires

## Abstract

**Background:**

Different questionnaires assess self-reported medication adherence and others quantify aspects of patients attitudes towards medication, but not together in a single instrument. Gathering these two aspects in a single instrument could reduce patients survey burden.

**Aim:**

The aim of this study was to develop the Medication Adherence Universal Questionnaire (MAUQ) using the Maastricht Utrecht Adherence in Hypertension short version (MUAH-16) factorial structure as the hypothesized model.

**Method:**

A multistep process started with the modification of the MUAH-16 to obtain the MAUQ. Patients using at least one antihypertensive medicine were recruited. The two questionnaires, the MUAH-16 and MAUQ, were applied. A confirmatory factor analysis (CFA) was performed using the initial MUAH-16 s-order 4-factor model. An additional bifactor model with four uncorrelated factors and an overall score was tested. The comparative fit index (CFI), root mean square error of approximation (RMSEA) with confidence intervals (CIs), and standardized root mean squared residual (SRMR) were used to assess both models.

**Results:**

A sample of 300 hypertensive patients completed the instruments. The CFA with the second-order 4-factor solution resulted in similar results for the MUAH-16 and MAUQ: CFIs of 0.934 and 0.930, RMSEAs of 0.043 [CI 0.030–0.056] and 0.045 [CI 0.031–0.057] and SRMRs of 0.060 and 0.061, respectively. The CFA with the bifactor model showed slightly better results for both the MUAH-16 and MAUQ: CFIs of 0.974 and 0.976, RMSEAs of 0.030 [CI 0.005–0.046] and 0.028 [CI 0.001–0.044], and SRMRs of 0.043 and 0.044, respectively.

**Conclusion:**

CFA demonstrated that the MAUQ presented a better fit to both models than the MUAH-16, obtaining a robust universal free instrument to assess medicine-taking behaviour and four medicine beliefs components.

**Supplementary Information:**

The online version contains supplementary material available at 10.1007/s11096-023-01612-x.

## Impact statements

- The Medication Adherence Universal Questionnaire (MAUQ®) is an open access universal medication adherence instrument that should be carefully translated into different languages.
- The MAUQ is an instrument used to assess medication-taking behaviour and provides four subscores representing four areas of beliefs about medication use: positive attitudes towards health care and medication, lack of discipline, aversion towards medication, and active coping with health problems.
- The performance of the MAUQ in different populations and appropriate cut-offs should be determined in future specific studies.

## Introduction

Medication adherence is a multifactorial and dynamic process shaped by several interacting factors, including personal, clinical and social variables [1, 2]. Based on this variability in contributing factors, the identification of patient determinants for nonadherence behaviour is recommended [3], and previous reports have shown better results when interventions are tailored to patient-specific nonadherence factors, especially for chronic medicines users [4–6].

Particular focus has been placed on the relationship between medication adherence and the beliefs that patients hold towards their medication. Intentional nonadherent patients hold beliefs that are significantly different from those of adherent and unintentional nonadherent patients [7]. The necessities-concerns framework was idealized to explain the influence of patients’ beliefs about medication and their intentional adherence behaviour [8]. A meta-analysis provided evidence about the value of this framework in predicting adherence to medication prescribed for chronic conditions [9].

The most widely used tool to elicit medication beliefs is the Beliefs about Medicines Questionnaire (BMQ) [8, 10]. The BMQ has two subscales: the necessity and concerns subscales. A high score on the necessity subscale suggests that an individual has strong beliefs in the necessity of their medicine, and a high score on the concerns subscale suggests that an individual has strong concerns about the negative effects of taking medicines. Scores from the BMQ have been correlated with medication adherence in several studies across patients with different conditions, with necessity beliefs associated with improved adherence behaviour and concern beliefs related to worse adherence results [8]. Although the BMQ was classified as assessing “beliefs associated with adherence”, this instrument was not conceptually developed to identify adherence, especially among potentially unintentional nonadherent patients [11].

A systematic review classified the 43 self-report adherence scales into five groups based on the information contained in the items used to construct the instruments [11]. However, the potential discriminatory power of the applicability to identify intentional and nonintentional nonadherent patients was not mentioned. The Maastricht Utrecht Adherence in Hypertension questionnaire (MUAH) was one of the instruments classified in the “barriers and beliefs” group because it provides health professionals with information about the causes of poor patient adherence to antihypertensive drugs [12]. The original MUAH is a 25-item questionnaire with four subscales: positive attitudes towards health care and medication (PAM), a lack of discipline (LD), aversion towards medication (ATM), and active coping with health problems (ACHP). However, the MUAH presents two limitations: the instrument lacks an overall score that allows adherent behaviour to be measured and is a disease-specific instrument [12].

To overcome the first of these limitations, a 16-item MUAH short version (MUAH-16) was created [13], first by eliminating the items with weaker contributions to the factor and then inverting the scores for the items constituting the subscales that were inversely associated with adherence, LD and ATM. The MUAH-16 maintains the four subscales of the MUAH that represent four areas of beliefs about medication taking. This modification allowed us to obtain an overall score representing medication-taking behaviour, which demonstrated the best fit in a confirmatory factor analysis (CFA). Concurrent validity of the MUAH-16 was demonstrated by correlation with the Medida de Adesão a Terapeutica (Measure of Treatment Adherence), an overall adherence behaviour questionnaire. Nevertheless, the MUAH-16 was only designed to assess adherence to antihypertensive drugs.

### Aim

The objective of the present study was to develop the Medication Adherence Universal Questionnaire (MAUQ) based on the latent variable structure of the MUAH-16.

### Ethics approval

The study was conducted in accordance with the Declaration of Helsinki and approved by the Ethics Committee of the Center Portugal Regional Health Administration (registration number: CE 73-2020).

## Method

### Instruments

The construction of a new universal instrument was designed through a multistep process that started with the analysis of the structure and content of the MUAH-16 by a multidisciplinary research team [13]. The text of the 6 items that specifically referred to blood pressure or hypertension (e.g., If I take my medication every day, I feel confident that my blood pressure is under control) were modified to be appropriate for any chronic disease (e.g., If I take my medication every day, I feel confident that my disease is under control). These 6 items were distributed in all the components except LD. At the end of this process, the Medication Adherence Universal Questionnaire—(MAUQ®) was created (Supplementary File 1). For testing purposes, a composite questionnaire merging both instruments (MUAH-16 + MAUQ) was also generated, resulting in a 22-item composite questionnaire. The complete evolution from the MUAH (25 items) to the MAUQ together with the differential characteristics of the instruments is presented in Table 1.

**Table 1:** Characteristics of the three instruments involved in the evolution to create the Medication Adherence Universal Questionnaire (MAUQ®)

Instrument	Created	Num. items	Overall score	Target population
MUAH	Wetzels et al. [12]	25	No	Hypertensive patients
MUAH-16	Cabral et al. [13]	16	Yes	Hypertensive patients
MAUQ	Cabral et al.	16	Yes	Chronic medicines users

*MUAH* Maastricht Utrecht Adherence in Hypertension, *MUAH-16* Maastricht Utrecht Adherence in Hypertension short version

### Study design

This was a cross-sectional study. No consensus exists about the minimum sample size required for CFA, ranging from 100 to 1000 patients [14], with 300 participants considered “good” [15]. Other authors prefer the ratio of the number of participants to the number of variables, with minimums ranging from 10 to 1 variable to 20 to 1 [16, 17]. A sample of 300 individuals was planned to widely satisfy both criteria for a 16-item questionnaire. Thus, a purposive sample of 300 patients aged over 18 years and taking at least one antihypertensive medicine (ATC classes C07, C08, and C09) was recruited from 3 community pharmacies in central Portugal between November 2020 and March 2021. Study aims and procedures were explained to potentially eligible patients. After signing an informed consent form, the composite questionnaire (MUAH-16 + MAUQ) was administered by a trained pharmacist, and sociodemographic and basic clinical data were collected.

### Data analysis

Overall and belief component scores of the two instruments were compared by means of a correlation analysis using Pearson’s correlation coefficient, R/R Studio (Posit Software, Boston, MA, USA) and the ggplot2 package [18]. Intrascale correlations were also assessed between the overall score and the beliefs component scores. Moderate to weak correlations were expected between the component and overall scores, but weak correlations should exist between the different components, demonstrating that they were all needed. Significance was established at the threshold of *p* < 0.05. To better evaluate the agreement between the scores of the two instruments throughout the range of potential results [19], both for the overall and the component scores, a Bland‒Altman analysis was also conducted using R/R Studio and the BlandAltmanLeh package [20]. Finally, to identify the adjustment of the MAUQ to the previously established latent structure of the MUAH-16, a CFA was conducted with the maximum likelihood method over the second-order 4-factor model previously reported for the MUAH-16 [13]. A second model, a bifactor model with the four uncorrelated latent variables and the overall score as a fifth latent variable, was also tested. CFAs were performed using R/R Studio and the lavaan package [21].

## Results

A total of 300 hypertensive medication users completed the composite questionnaire, with a mean age of 68.6 years (SD 9.9); 53.7% of the participants were women. The mean number of years since hypertension diagnosis was 14.3 years (SD 9.9), and participants used 4.3 medicines on average (SD 2.6). The most frequently reported comorbidities included dyslipidaemia (179 participants; 59.7%), obesity (123 participants; 41.0%), diabetes (62 participants; 20.7%), and chronic obstructive pulmonary disease (40 participants; 13.3%).

The results obtained for each of the instruments are presented in Supplementary File 2, and the overall scores, as well as the component scores, for both instruments are shown in Table 2. Cronbach’s alpha was 0.551 for the overall MUAH-16 score and 0.569 for the MAUQ score. Cronbach’s alphas for the belief components of the MUAH-16 and MAUQ were 0.673 and 0.637 for the PAM subscale, 0.777 for the LD subscale (both instruments), 0.660 and 0.694 for the ATM subscale, and 0.627 and 0.539 for the ACHP subscale, respectively.

**Table 2:** Scores obtained for the two questionnaires, the Maastricht Utrecht Adherence in Hypertension short version (MUAH-16) and the Medication Adherence Universal Questionnaire (MAUQ®)

N = 300	MUAH-16 mean (SD)	MAUQ mean (SD)	Pearson’s r (95% CI)
Overall score	83.37 (8.85)	84.09 (8.91)	0.946 (0.933 : 0.957)
Positive attitude towards health care and medication	25.49 (2.49)	25.68 (2.23)	0.846 (0.810 : 0.875)
Lack of discipline	23.88 (4.69)	23.88 (4.69)	–
Aversion towards medication	15.64 (5.51)	15.46 (5.75)	0.963 (0.953 : 0.970)
Active coping with health problems	18.36 (4.40)	19.06 (3.98)	0.855 (0.821 : 0.883)

*All correlations were significant at the 0.001 level

The correlation analyses (Supplementary File 3) for the overall score and the three belief component scores with text modifications showed a high concordance. Table 3 presents the intrascale correlation coefficients between the overall score and the beliefs component scores for each of the instruments, resulting in medium to strong correlations between the overall score and the beliefs component scores of each instrument but weak or null correlations between each instrument’s components.

**Table 3:** Intra-scale correlations between the overall score and beliefs components of both instruments the Maastricht Utrecht Adherence in Hypertension short version (MUAH-16) and the Medication Adherence Universal Questionnaire (MAUQ®)

Instrument	Component	Overall	PAM	LD	ATM	ACHP
MUAH-16	Overall	1				
	PAM	0.442	1			
	LD	0.631	0.148	1		
	ATM	0.707	0.149	0.306	1	
	ACHP	0.203	ns	− 0.263	− 0.240	1
MAUQ	Overall	1				
	PAM	0.439	1			
	LD	0.651	0.135	1		
	ATM	0.742	0.153	0.326	1	
	ACHP	0.155	ns	− 0.267	− 0.252	1

*PAM* positive attitude towards health care and medication, *LD* lack of discipline, *ATM* aversion towards medication, *ACHP* active coping with health problems, *ns* not significant (*p* > 0.05)

**Fig. 1:**
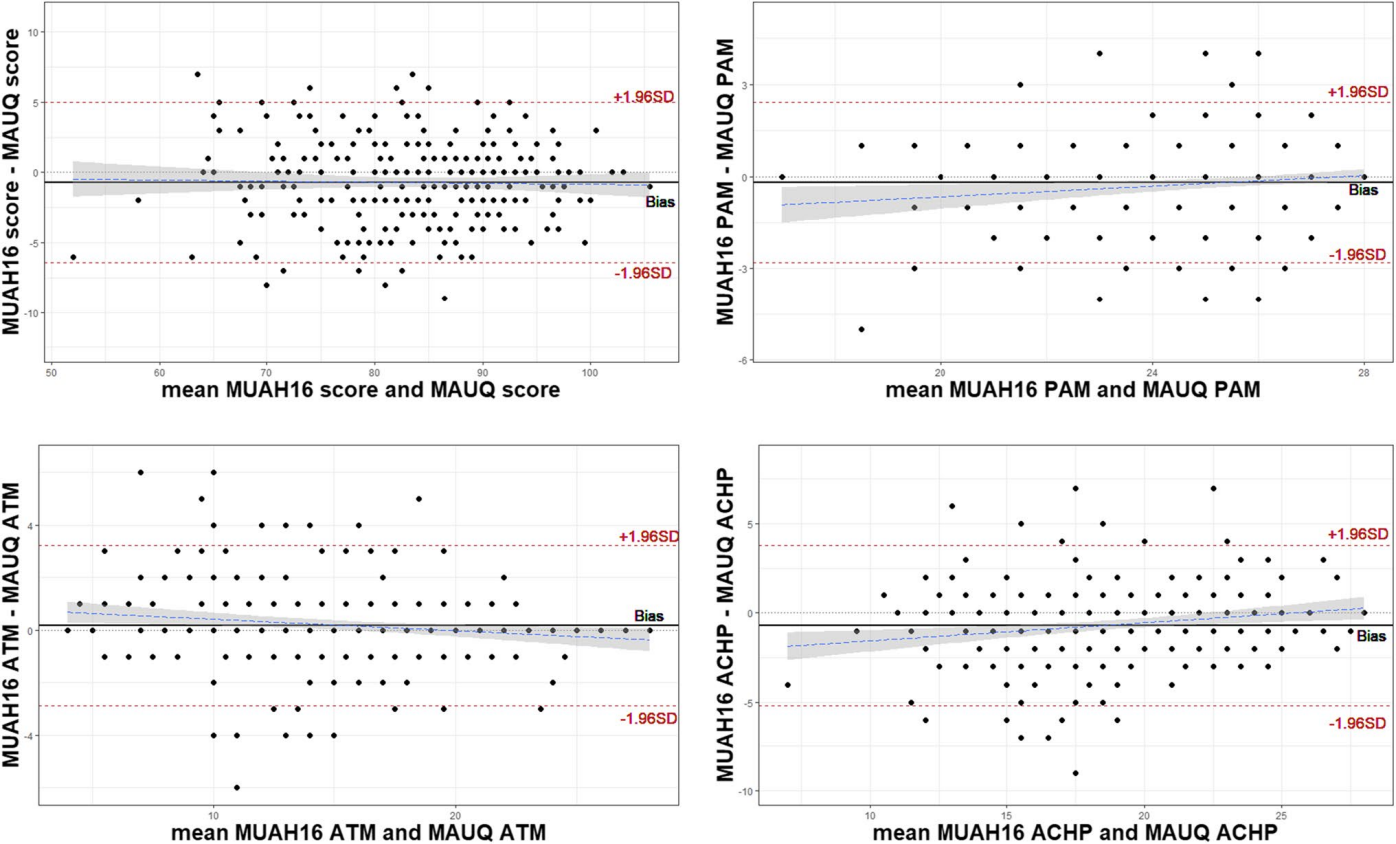
Bland-Altman analyses between Maastricht Utrecht Adherence in Hypertension short version (MUAH-16) and the Medication Adherence Universal Questionnaire (MAUQ®) overall and beliefs component scores. Note: Analysis for the ‘Lack of Discipline’ component was omitted because items were identical in both instruments

Bland‒Altman plots (Fig. 1) for the overall and beliefs component scores of both instruments demonstrated that, for all comparisons, differences between the two instruments were between the expected limits, and no trends existed throughout the result intervals.

**Fig. 2:**
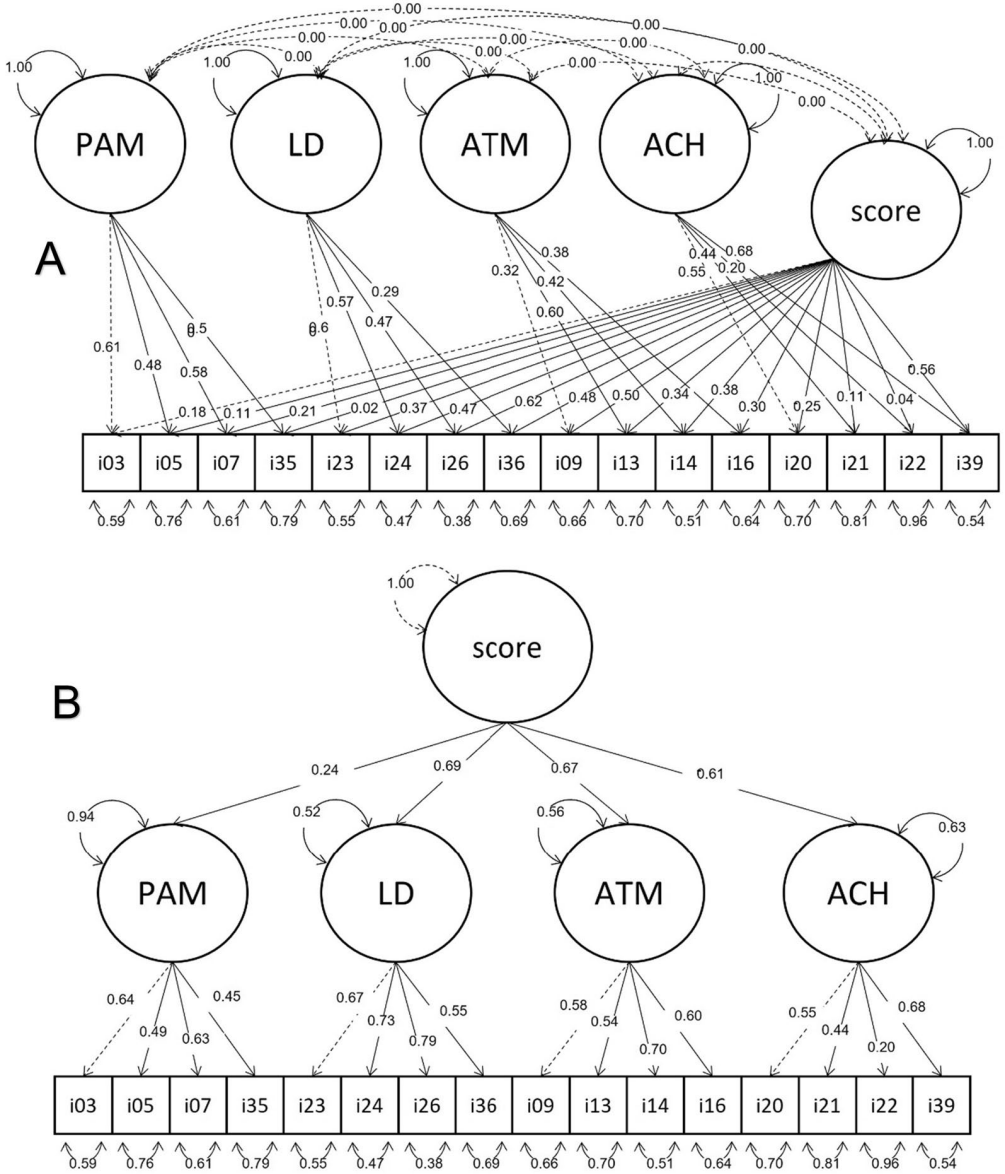
Confirmatory factor analysis of the Medication Adherence Universal Questionnaire (MAUQ®) using a second order 4-factor model (**A**) and using a bifactor model with the four factors uncorrelated (**B**)

CFAs were run for the two models (i.e., the second order 4-factor model—Fig. 2A, and the bifactor model with the four uncorrelated factors—Fig. 2B) and demonstrated that the MAUQ and MUAH-16 presented similar results (Table 4). The results for both models were also very similar, with a slightly better standardized root mean square residual (SRMS) in the bifactor model. This model also had a lower number of degrees of freedom, which resulted in a greater difference from the second-order 4-factor model when parsimony correction was applied to fit indices, presenting lower root mean square errors of approximation (RMSEAs) for both instruments. Finally, the high comparative fit index (CFI) of the two models for both instruments showed a good fit with both user-specified models, with better results in the bifactor model.

**Table 4:** Comparison of the model fit for Maastricht Utrecht Adherence in Hypertension short version (MUAH-16) and the Medication Adherence Universal Questionnaire (MAUQ®)

	Second order 4-factor model		Bifactor model	
	MAUQ	MUAH-16	MAUQ	MUAH-16
χ^2^	159.913	156.761	108.645	111.873
Degrees of freedom	100	100	88	88
CFI	0.930	0.934	0.976	0.974
RMSEA (CI)	0.045 (0.031 : 0.057)	0.043 (0.030 : 0.056)	0.028 (0.001 : 0.044)	0.030 (0.005 : 0.046)
SRMR	0.061	0.060	0.044	0.043

*CFI* comparative fit index, *CI* confidence interval, *RMSEA* root mean square error of approximation, *SRMR* standardized root mean squared residual

## Discussion

### Statement of key findings

With the intention to serve as a universal freely available medication adherence assessment instrument, the MAUQ® was created not only to assess overall adherence behaviour but also to comparatively weight four beliefs components that may lead to nonadherence.

### Strengths and weaknesses

The use of universal instruments facilitates interstudy comparability and enables quantitative evidence synthesis through meta-analyses. However, poor reporting practices are common in health studies. Data omissions are frequently associated with bias towards favouring drugs [22]. Several guidelines have been created to direct how different study designs should be reported. In the adherence field, specific guidelines [23] exist to appropriately report the different elements of the adherence process [24]. Unfortunately, these guidelines are not always sufficient to eliminate poor reporting practices [25, 26]. With the ultimate goal of improving comparability and facilitating quantitative evidence synthesis, the MAUQ is freely available to any researcher or practitioner once fair use conditions are accepted. These conditions comprise two requirements: (a) ensuring consistency among different versions of the instrument and (b) ensuring complete reporting practices (Supplementary File 1).

Some limitations may affect this study. The MAUQ was developed in the European Portuguese language, and its psychometric properties should be confirmed in other languages or language variations. To ensure a robust validation process, the sample included to validate the instrument consisted of a population of hypertensive patients. Further analyses of the performance of the MAUQ in other conditions must be performed to ensure the universal character of the instrument. A cut-off to discriminate between adherent and nonadherent patients cannot be established until results are obtained for convergent validity analyses with objective/direct medication adherence methods.

### Interpretation and further research

Several guidelines have been created to adequately analyse and report patient-reported outcomes (PROM) and patient-reported experiences (PREM) [27, 28]. Over the years, many self-report adherence scales have been developed. However, no general agreement exists on the best instrument [29, 30]. Some instruments are disease specific, which limits their use in broad comparisons or for pharmacoeconomic studies. Other instruments have restrictive use conditions, which is against the open science paradigm and has resulted in abusive practices [31]. Most of these instruments evaluate only medicine-taking behaviour or barriers and beliefs about medication use separately but very rarely evaluate both together.

Using two instruments at the same time (i.e., adherence behaviour plus beliefs about medicines) increases the number of questions and subsequently increases the respondent burden due to potential response fatigue. In fact, applying any self-response instrument during a patient-provider encounter is subject to the three categories of respondent burden [32]: “distrust”, “distress” and “cognitive burden”. The latter may depend not only on the complexity of the questions but also on the repetitiveness of the questions, which may occur when applying two related instruments. However, reducing the survey length should be done carefully while considering the content of the instrument rather than the length itself [33]. During this multistep process, we first reduced the length of the MUAH (25 items) to 16 items, inverted the scores of four items into two factors resulting in the MUAH-16, and modified six items to obtain a general, not disease-specific, (i.e., universal) instrument: the MAUQ. After this process, the MAUQ was shown to have good psychometric properties, with a latent variable structure appropriate to assess both overall adherence and each of the four beliefs about medicines. Future research should establish the adequate performance of the MAUQ in different conditions and populations, as well as the appropriate nonadherence discriminant cut-off for the overall score and potential ratios between the different components to better tailor adherence-enhancing interventions.

## Conclusion

CFA demonstrated that the MAUQ presented a better fit to both latent variable models than the MUAH-16, obtaining a robust instrument to assess medicine-taking behaviour and four medicine beliefs components. An intensive assessment of the performance and convergent validity of the MAUQ in different populations and medical conditions, as well as cross-cultural adaptation to different languages, has been initiated to create a universal open access (freely available) medication adherence instrument.

### Supplementary Information

Below is the link to the electronic supplementary material.

Supplementary file1 (PDF 409 KB)

Supplementary file2 (PDF 140 KB)

Supplementary file3 (PDF 214 KB)

## References

[1] World Health Organization. Adherence to long-term therapies—evidence for action 2003. https://apps.who.int/iris/bitstream/handle/10665/42682/9241545992.pdf. Accessed 15 Dec 2022.

[2] Sawalha R, Hosseinzadeh H, Bajorek B. Culturally and linguistically diverse patients’ perspectives and experiences on medicines management in Australia: a systematic review. Int J Clin Pharm. 2023. 10.1007/s11096-023-01560-6. 37020057 10.1007/s11096-023-01560-6

[3] Allemann SS, van den Nieuwlaat R, et al. Matching adherence interventions to patient determinants using the theoretical domains Framework. Front Pharmacol. 2016;7:429. 10.3389/fphar.2016.00429. 27895583 10.3389/fphar.2016.00429 PMC5107738

[4] Gregoriano C, Dieterle T, Breitenstein AL, et al. Does a tailored intervention to promote adherence in patients with chronic lung disease affect exacerbations? A randomized controlled trial. Respir Res. 2019;20(1):273. 10.1186/s12931-019-1219-3. 31796013 10.1186/s12931-019-1219-3 PMC6892023

[5] Xu HY, Yu YJ, Zhang QH, et al. Tailored interventions to improve medication adherence for cardiovascular diseases. Front Pharmacol. 2020;11:510339. 10.3389/fphar.2020.510339. 33364935 10.3389/fphar.2020.510339 PMC7751638

[6] Livori AC, Pol D, Levkovich B, et al. Optimising adherence to secondary prevention medications following acute coronary syndrome utilising telehealth cardiology pharmacist clinics: a matched cohort study. Int J Clin Pharm. 2023. 10.1007/s11096-023-01562-4. 36940081 10.1007/s11096-023-01562-4 PMC10026199

[7] Clifford S, Barber N, Horne R. Understanding different beliefs held by adherers, unintentional nonadherers, and intentional nonadherers: application of the necessity-concerns Framework. J Psychosom Res. 2008;64(1):41–6. 10.1016/j.jpsychores.2007.05.004. 18157998 10.1016/j.jpsychores.2007.05.004

[8] Horne R, Weinman J. Patients’ beliefs about prescribed medicines and their role in adherence to treatment in chronic physical illness. J Psychosom Res. 1999;47(6):555–67. 10.1016/s0022-3999(99)00057-4. 10661603 10.1016/s0022-3999(99)00057-4

[9] Horne R, Chapman SC, Parham R, et al. Understanding patients’ adherence-related beliefs about medicines prescribed for long-term conditions: a meta-analytic review of the necessity-concerns Framework. PLoS ONE. 2013;8(12):e80633. 10.1371/journal.pone.0080633. 24312488 10.1371/journal.pone.0080633 PMC3846635

[10] Salgado T, Marques A, Geraldes L, et al. Cross-cultural adaptation of the beliefs about Medicines Questionnaire into Portuguese. Sao Paulo Med J. 2013;131(2):88–94. 10.1590/s1516-31802013000100018. 23657510 10.1590/s1516-31802013000100018 PMC10871720

[11] Nguyen TM, La Caze A, Cottrell N. What are validated self-report adherence scales really measuring? A systematic review. Br J Clin Pharmacol. 2014;77(3):427–45. 10.1111/bcp.12194. 23803249 10.1111/bcp.12194 PMC3952718

[12] Wetzels G, Nelemans P, van Wijk B, et al. Determinants of poor adherence in hypertensive patients: development and validation of the “Maastricht Utrecht Adherence in Hypertension (MUAH)-questionnaire. Patient Educ Couns. 2006;64(1–3):151–8. 10.1016/j.pec.2005.12.010. 16427764 10.1016/j.pec.2005.12.010

[13] Cabral AC, Castel-Branco M, Caramona M, et al. Developing an adherence in hypertension questionnaire short version: MUAH-16. J Clin Hypertens (Greenwich). 2018;20(1):118–24. 10.1111/jch.13137. 29171719 10.1111/jch.13137 PMC8031096

[14] Kline P. An easy guide to factor analysis. New York: Routledge; 1994. ISBN: 9780415094900.

[15] Comrey AL, Lee HB. A first course in factor analysis. Hillsdale: Lawrence Erlbaum; 1992. ISBN: 9781315827506.

[16] Everitt BS. Multivariate analysis: the need for data, and other problems. Br J Psychiatry. 1975;126:237–40. 10.1192/bjp.126.3.237. 1125504 10.1192/bjp.126.3.237

[17] Hair JF, Anderson RE, Tatham RL et al. Multivariate data analysis. Saddle River: Prentice Hall; 1995. ISBN: 9780139133107.

[18] Wickham H. ggplot2: elegant graphics for data analysis. New York: Springer; 2016. ISBN: 978-0-387-98141-3.

[19] Bland JM, Altman DG. Statistical methods for assessing agreement between two methods of clinical measurement. Lancet. 1986;1(8476):307–10. 10.1016/S0140-6736(86)90837-8. 2868172 10.1016/S0140-6736(86)90837-8

[20] Lehnert B. BlandAltmanLeh: Plots. https://cran.r-project.org/web/packages/BlandAltmanLeh/index.html. Accessed 15 Dec 2022.

[21] Rosseel Y. lavaan: an R package for structural equation modeling. J Stat Softw. 2012;48(2):1–36. 10.18637/jss.v048.i02. 10.18637/jss.v048.i02

[22] Glasziou P, Altman DG, Bossuyt P, et al. Reducing waste from incomplete or unusable reports of biomedical research. Lancet. 2014;383(9913):267–76. 10.1016/S0140-6736(13)62228-X. 24411647 10.1016/S0140-6736(13)62228-X

[23] De Geest S, Zullig LL, Dunbar-Jacob J, et al. ESPACOMP medication adherence reporting guideline (EMERGE). Ann Intern Med. 2018;169(1):30–5. 10.7326/M18-0543. 29946690 10.7326/M18-0543 PMC7643841

[24] Vrijens B, De Geest S, Hughes DA, et al. A new taxonomy for describing and defining adherence to medications. Br J Clin Pharmacol. 2012;73(5):691–705. 10.1111/j.1365-2125.2012.04167.x. 22486599 10.1111/j.1365-2125.2012.04167.x PMC3403197

[25] Turner L, Shamseer L, Altman DG, et al. Consolidated standards of reporting trials (CONSORT) and the completeness of reporting of randomised controlled trials (RCTs) published in medical journals. Cochrane Database Syst Rev. 2012;11(11):MR000030. 10.1002/14651858.MR000030.pub2. 23152285 10.1002/14651858.MR000030.pub2 PMC7386818

[26] Tonin FS, Borba HH, Leonart LP, et al. Methodological quality assessment of network meta-analysis of drug interventions: implications from a systematic review. Int J Epidemiol. 2019;48(2):620–32. 10.1093/ije/dyy197. 30212868 10.1093/ije/dyy197

[27] Mokkink LB, de Vet HCW, Prinsen CAC, et al. COSMIN risk of bias checklist for systematic reviews of patient-reported outcome measures. Qual Life Res. 2018;27(5):1171–9. 10.1007/s11136-017-1765-4. 29260445 10.1007/s11136-017-1765-4 PMC5891552

[28] Bull C, Byrnes J, Hettiarachchi R, et al. A systematic review of the validity and reliability of patient-reported experience measures. Health Serv Res. 2019;54(5):1023–35. 10.1111/1475-6773.13187. 31218671 10.1111/1475-6773.13187 PMC6736915

[29] Lavsa SM, Holzworth A, Ansani NT. Selection of a validated scale for measuring medication adherence. J Am Pharm Assoc. 2011;51(1):90–4. 10.1331/JAPhA.2011.09154. 10.1331/JAPhA.2011.09154 21247831

[30] Culig J, Leppee M. From Morisky to Hill-bone; self-reports scales for measuring adherence to medication. Coll Antropol. 2014;38(1):55–62. 24851597

[31] Azeredo TB, Reis RD, Fernandez-Llimos F. Scales and questionnaires under copyright: additional barriers to health research. Rev Bras Farm Hosp Serv Saude. 2021;12(1):610. 10.30968/rbfhss.2021.121.0610. 10.30968/rbfhss.2021.121.0610

[32] Wenemark M, Hollman Frisman G, Svensson T, et al. Respondent satisfaction and respondent burden among differently motivated participants in a health-related survey. Field Methods. 2010;22(4):378–90. 10.1177/1525822X10376704. 10.1177/1525822X10376704

[33] Rolstad S, Adler J, Ryden A. Response burden and questionnaire length: is shorter better? A review and meta-analysis. Value Health. 2011;14(8):1101–8. 10.1016/j.jval.2011.06.003. 22152180 10.1016/j.jval.2011.06.003

