# Correction: Development and validation of a Medication Adherence Universal Questionnaire: the MAUQ

**DOI:** 10.1007/s11096-024-01795-x

**Published:** 2024-08-27

**Authors:** Ana C. Cabral, Marta Lavrador, Margarida Castel-Branco, Isabel Vitória Figueiredo, Fernando Fernandez-Llimos

**Affiliations:** 1 Pharmacology and Pharmaceutical Care Laboratory, Faculty of Pharmacy, University of Coimbra, Coimbra, Portugal; 2 Institute for Clinical and Biomedical Research (iCBR), Faculty of Medicine, University of Coimbra, Coimbra, Portugal; 3 Laboratory of Pharmacology, Department of Drug Sciences, Faculty of Pharmacy, University of Porto, Porto, Portugal; 4 Applied Molecular Biosciences (UCIBIO), University of Porto, Porto, Portugal

**Correction to: International Journal of Clinical Pharmacy (2023) 45:999–1006** 10.1007/s11096-023-01612-x

The wrong Supplementary file (Supplementary file 1) was originally published with this article; it has now been replaced with the correct file.

The original article has been corrected.

## Supplementary Information

Below is the link to the electronic supplementary material.

Supplementary file1 (PDF 409 KB)

