# Supplementary material for: Development and validation of a Medication Adherence Universal Questionnaire: the MAUQ
Source: Int J Clin Pharm. 2023 Jun 17;45(4):999–1006. doi: 10.1007/s11096-023-01612-x (PMC10366321; doi:10.1007/s11096-023-01612-x)
Supplement: Supplementary file 1 — Supplementary file1 (PDF 409 KB) [file 11096_2023_1612_MOESM1_ESM.pdf]

Medication Adherence Universal Questionnaire (MAUQ-pt-PT)

Validated Portugal Portuguese version of the Medication Adherence Universal Questionnaire (MAUQ-pt-PT).

Aiming to safeguard the consistency of their psychometric properties, MAUQ® and all MAUQ® versions are copyrighted by the original research team. However, these instruments were created with the intention of being free available to any researcher or practitioner under fair use conditions:

#1. Cross-culturally adapting a new version of the MAUQ®

To create a new version of the MAUQ®, adapted to any language or language variation, a cross-cultural adaptation process should be conducted following ISPOR standards. To ensure the consistency with the original version and among all other language versions, the two research teams (i.e., the new language team and the original research team) should be conjointly involved in the new version validation process. The cross-culturally adapted version of the MAUQ must be approved by the original research team, and conjointly published. The cross-culturally adapted version of the MAUQ must be free available after publication, without any embargo, following the fair use conditions. A copy of this MAUQ fair use policy must be included in the validation article or appendixes.

MAUQ® versions should be recognized by using a standard coding system based on the combination of ISO 639-1 codes[1] for the language abbreviation, and the ISO 3166 alpha-2 codes[2] for the country (language variation). For example, Portugal Portuguese version of the MAUQ® is abbreviated as MAUQ-pt-PT, while American English version should be abbreviated as MAUQ-en-US.

#2. Using a validated version of the MAUQ®

MAUQ® was created to be free available to any researcher or practitioner, once a validated and published language version existed. Researchers or practitioners do not have to ask for permission to use for research or practice purposes any published version of the MAUQ®. No modifications are allowed to any published version of the MAUQ®. When a study using the MAUQ is published, the original MAUQ® and the version validation articles must be referenced.

#3. Reporting the results of a MAUQ® study

Ensuring replicability is a major concern of the MAUQ research team. Literature demonstrated that poor reporting practices hamper replicability. All the studies reporting MAUQ® results must, at least, provide: a) mean and standard deviation of the MAUQ® overall score, b) means and standard deviations of the scores of the four MAUQ® components (i.e., PAM: positive attitudes towards health care and medication; LD: lack of discipline; ATM: aversion towards medication; ACHP: active coping with health problems); c) a table with the response frequencies of each MAUQ® item (can be in appendix).

---

[1] https://en.wikipedia.org/wiki/List_of_ISO_639-1_codes
[2] https://en.wikipedia.org/wiki/List_of_ISO_3166_country_codes

Medication Adherence Universal Questionnaire (MAUQ-pt-PT)®

			Discordo totalmente →						Concordo totalmente
APM	3	Sinto-me melhor ao tomar a medicação todos os dias	1	2	3	4	5	6	7
APM	5	Se eu tomar a minha medicação todos os dias, acredito que a minha doença está sob controlo	1	2	3	4	5	6	7
APM	7	As vantagens de tomar a medicação pesam mais do que as desvantagens	1	2	3	4	5	6	7
AM	9	Quando a minha doença está sob controlo na consulta médica, eu quero tomar menos medicamentos	7	6	5	4	3	2	1
AM	13	Não gosto de tomar medicamentos todos os dias	7	6	5	4	3	2	1
AM	14	Tenho medo dos efeitos secundários	7	6	5	4	3	2	1
AM	16	Penso que não é saudável para o organismo tomar medicamentos todos os dias	7	6	5	4	3	2	1
APPS	20	Tenho o cuidado de fazer exercício suficiente para cuidar da minha saúde	1	2	3	4	5	6	7
APPS	21	Faço uma alimentação saudável para cuidar da minha saúde	1	2	3	4	5	6	7
APPS	22	Evito comportamentos que podem prejudicar a minha saúde (ex. tabaco, álcool)	1	2	3	4	5	6	7
FD	23	Às vezes não tenho a certeza se tomei os meus medicamentos	7	6	5	4	3	2	1
FD	24	Tenho uma vida agitada; é por isso que às vezes me esqueço de tomar a minha medicação	7	6	5	4	3	2	1
FD	26	Durante as férias, ou fins de semana, às vezes esqueço-me de tomar a minha medicação	7	6	5	4	3	2	1
APM	35	Penso que contribuo para uma melhoria da minha doença quando tomo os medicamentos todos os dias	1	2	3	4	5	6	7
FD	36	Acho difícil cumprir o meu esquema diário de toma de medicação	7	6	5	4	3	2	1
APPS	39	Reúno informação sobre as possibilidades de resolver problemas de saúde	1	2	3	4	5	6	7

APM: atitudes positivas em relação aos medicamentos e cuidados de saúde; FD: Falta de disciplina; AM: Aversão à medicação; APPS: Atitudes proactivas em relação aos problemas de saúde.

See the conditions to use the MAUQ® at the first page of this document.

Unvalidated translation of the Medication Adherence Universal Questionnaire (MAUQ)

			Completely disagree			→			Completely agree
PAM	3	I feel better taking medication every day	1	2	3	4	5	6	7
PAM	5	If I take my medicines every day, I think my disease is under control	1	2	3	4	5	6	7
PAM	7	The pros of taking medication weight up against the cons	1	2	3	4	5	6	7
ATM	9	When my disease is under control during my medical checkups, I want to take less medication	1	2	3	4	5	6	7
ATM	13	I dislike taking medication every day	1	2	3	4	5	6	7
ATM	14	I am afraid of side effects	1	2	3	4	5	6	7
ATM	16	I think it is not healthy for your body to take medication every day	1	2	3	4	5	6	7
ACPH	20	I take special care to do enough exercise to take care of my health	1	2	3	4	5	6	7
ACPH	21	I eat healthy to take care of my health	1	2	3	4	5	6	7
ACPH	22	I avoid behaviors that can harm my health (e.g., tobacco, alcohol)	1	2	3	4	5	6	7
LD	23	It happens that I am not sure whether I have taken my tablets	1	2	3	4	5	6	7
LD	24	I have a busy life, that is why I sometimes forget to take my medication	1	2	3	4	5	6	7
LD	26	During holidays or weekends, I sometimes forget to take my medication	1	2	3	4	5	6	7
PAM	35	I think I contribute to the improvement of my disease when I take my medication every day	1	2	3	4	5	6	7
LD	36	I find it hard to stick to my daily regimen of medication taking	1	2	3	4	5	6	7
ACPH	39	I gather information about possibilities to solve health problems	1	2	3	4	5	6	7

PAM: positive attitudes towards health care and medication; LD: lack of discipline; ATM: aversion towards medication; ACHP: active coping with health problems.

See the conditions to use the MAUQ® at the first page of this document.

Unvalidated
