# Supplementary material for: Development and validation of a Medication Adherence Universal Questionnaire: the MAUQ
Source: Int J Clin Pharm. 2023 Jun 17;45(4):999–1006. doi: 10.1007/s11096-023-01612-x (PMC10366321; doi:10.1007/s11096-023-01612-x)
Supplement: Supplementary file 2 — Supplementary file2 (PDF 140 KB) [file 11096_2023_1612_MOESM2_ESM.pdf]

Cabral AC, Lavrador M, Castel-Branco M, Figueiredo IV, Fernandez-Llimos F. Development and validation of a Medication Adherence Universal Questionnaire: The MAUQ

**Results for the MUAH-16**		Patients Answers						
		Totally disagree	Disagree	Somewhat disagree	Neither agree nor disagree	Somewhat agree	Agree	Totally agree
3	I feel better taking medication every day	0	1 (0,3%)	1 (0,3%)	8 (2,7%)	43 (14,3%)	85 (28,3%)	162 (54,0%)
5	If I take my medication every day, I feel confident that my blood pressure is under control	0	2 (2,7%)	2 (2,7%)	9 (3,0%)	31 (10,3%)	81 (27,0%)	175 (58,3%)
7	The pros of taking medication weight up against the cons	0	0	1 (0,3%)	16 (5,3%)	27 (9,0%)	80 (26,7%)	176 (58,7%)
9	When my blood pressure is under control during my medical checkups, I want to take less medication	85 (28,3%)	42 (14,0%)	39 (13,0%)	50 (16,7%)	35 (11,7%)	25 (8,3%)	24 (8,0%)
13	I dislike taking medication every day	19 (6,3%)	16 (5,3%)	19 (6,3%)	114 (38,0%)	36 (12,0%)	28 (9,3%)	68 (22,7%)
14	I am afraid of side effects	61 (20,3%)	41 (13,7%)	20 (6,7%)	32 (10,7%)	52 (17,3%)	38 (12,7%)	56 (18,7%)
16	I think it is not healthy for your body to take medication every day	31 (10,3%)	32 (10,7%)	24 (8,0%)	47 (15,7%)	72 (24,0%)	42 (14,0%)	52 (17,3%)
20	I take special care to do enough exercise to reduce the risk of getting cardiovascular diseases	17 (5,7%)	48 (16,0%)	50 (16,7%)	54 (18,0%)	68 (22,7%)	36 (12,0%)	27 (9,0%)
21	I eat less fat in order to avoid cardiovascular diseases	6 (2,0%)	6 (2,0%)	9 (3,0%)	41 (13,7%)	70 (23,3%)	92 (30,7%)	76 (25,3%)
22	I eat less salt in order to avoid cardiovascular diseases	1 (0,3%)	5 (1,7%)	9 (3,0%)	39 (13,0%)	65 (21,7%)	102 (34,0%)	79 (26,3%)
23	It happens that I am not sure whether I have taken my tablets	109 (36,3%)	104 (34,7%)	30 (10,0%)	12 (4,0%)	27 (9,0%)	12 (4,0%)	6 (2,0%)
24	I have a busy life; that is why I sometimes forget to take my medication	174 (58,0%)	48 (16,0%)	29 (9,7%)	10 (3,3%)	15 (5,0%)	12 (4,0%)	12 (4,0%)
26	During holidays or weekends I sometimes forget to take my medication	185 (61,7%)	59 (19,7%)	23 (7,7%)	4 (1,3%)	15 (5,0%)	8 (2,7%)	6 (2,0%)
35	I think I contribute to the improvement of my blood pressure when I take my medication every day	0	0	3 (1,0%)	7 (2,3%)	27 (9,0%)	89 (29,7%)	174 (58,0%)
36	I find it hard to stick to my daily regimen of medication taking	177 (59,0)	59 (19,7%)	30 (10,0%)	14 (4,7%)	12 (4,0%)	6 (2,0%)	2 (0,7%)
39	I gather information about possibilities to solve health problems	80 (26,7%)	64 (21,3%)	48 (16,0%)	17 (5,7%)	36 (12,0%)	27 (9,0%)	28 (9,3%)

MUAH-16: Maastricht Utrecht Adherence in Hypertension short version

Cabral AC, Lavrador M, Castel-Branco M, Figueiredo IV, Fernandez-Llimos F. Development and validation of a Medication Adherence Universal Questionnaire: The MAUQ

**Results for the MAUQ-pt-PT**		Patients Answers						
		Totally disagree	Disagree	Somewhat disagree	Neither agree nor disagree	Somewhat agree	Agree	Totally agree
3	I feel better taking medication every day	0	1 (0,3%)	1 (0,3%)	8 (2,7%)	43 (14,3%)	85 (28,3%)	162 (54,0%)
5	If I take my medication every day, I feel confident that my disease is under control	0	1 (0,3%)	0	5 (1,7%)	41 (13,7%)	103 (34,3%)	150 (50,0%)
7	The pros of taking medication weight up against the cons	0	0	1 (0,3%)	16 (5,3%)	27 (9,0%)	80 (26,7%)	176 (58,7%)
9	When my disease is under control during my medical checkups, I want to take less medication	84 (28,0%)	45 (15,0%)	23 (7,7%)	45 (15,0%)	42 (14,0%)	28 (9,3%)	33 (11,0%)
13	I dislike taking medication every day	19 (6,3%)	16 (5,3%)	19 (6,3%)	114 (38,0%)	36 (12,0%)	28 (9,3%)	68 (22,7%)
14	I am afraid of side effects	61 (20,3%)	41 (13,7%)	20 (6,7%)	32 (10,7%)	52 (17,3%)	38 (12,7%)	56 (18,7%)
16	I think it is not healthy for your body to take medication every day	31 (10,3%)	32 (10,7%)	24 (8,0%)	47 (15,7%)	72 (24,0%)	42 (14,0%)	52 (17,3%)
20	I take special care to do enough exercise to take care of my health	25 (8,3%)	54 (18,0%)	45 (15,0%)	59 (19,7%)	57 (19,0%)	36 (12,0%)	24 (8,0%)
21	I eat healthy to take care of my health	1 (0,3%)	0	14 (4,7%)	36 (12,0%)	83 (27,7%)	95 (31,7%)	71 (23,7%)
22	I avoid behaviors that can harm my health (eg, tobacco, alcohol)	2 (0,7%)	2 (0,7%)	3 (1,0%)	9 (3,0%)	23 (7,7%)	74 (24,7%)	187 (62,3%)
23	It happens that I am not sure whether I have taken my medication	109 (36,3%)	104 (34,7%)	30 (10,0%)	12 (4,0%)	27 (9,0%)	12 (4,0%)	6 (2,0%)
24	I have a busy life; that is why I sometimes forget to take my medication	174 (58,0%)	48 (16,0%)	29 (9,7%)	10 (3,3%)	15 (5,0%)	12 (4,0%)	12 (4,0%)
26	During holidays or weekends I sometimes forget to take my medication	185 (61,7%)	59 (19,7%)	23 (7,7%)	4 (1,3%)	15 (5,0%)	8 (2,7%)	6 (2,0%)
35	I think I contribute to the improvement of my disease when I take my medication every day	0	0	1 (0,3%)	2 (0,7%)	13 (4,3%)	64 (21,3%)	220 (73,3%)
36	I find it hard to stick to my daily regimen of medication taking	177 (59,0)	59 (19,7%)	30 (10,0%)	14 (4,7%)	12 (4,0%)	6 (2,0%)	2 (0,7%)
39	I gather information about possibilities to solve health problems	80 (26,7%)	64 (21,3%)	48 (16,0%)	17 (5,7%)	36 (12,0%)	27 (9,0%)	28 (9,3%)

MAUQ: Medication Adherence Universal Questionnaire
