# Supplementary material for: Development and validation of a Medication Adherence Universal Questionnaire: the MAUQ
Source: Int J Clin Pharm. 2023 Jun 17;45(4):999–1006. doi: 10.1007/s11096-023-01612-x (PMC10366321; doi:10.1007/s11096-023-01612-x)
Supplement: Supplementary file 3 — Supplementary file3 (PDF 214 KB) [file 11096_2023_1612_MOESM3_ESM.pdf]

Cabral AC, Lavrador M, Castel-Branco M, Figueiredo IV, Fernandez-Llimos F. Development and validation of a Medication Adherence Universal Questionnaire: The MAUQ

Correlation analyses between Maastricht Utrecht Adherence in Hypertension short version (MUAH-16) and the Medication Adherence Universal Questionnaire (MAUQ®) overall and beliefs component scores.

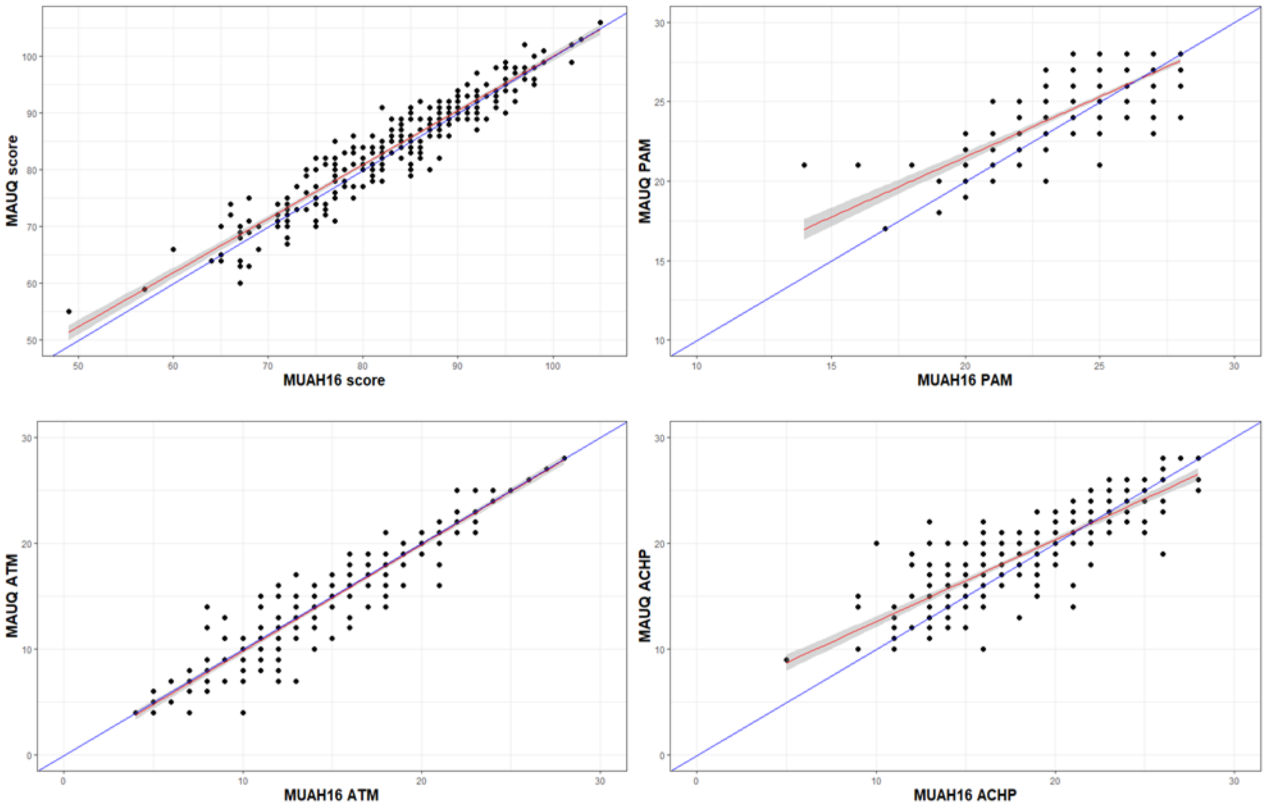

Note: Analysis for the Lack of Discipline component was omitted because items were identical in both instruments.

Correlation coefficients between overall score and beliefs subscales of Medication Adherence Universal Questionnaire (MAUQ®) and the Maastricht Utrecht Adherence in Hypertension short version (MUAH-16)			
N=300	MUAH-16 Mean (SD)	MAUQ Mean (SD)	Pearson's r (95%CI)
Overall score	83.37 (8.85)	84.09 (8.91)	0.946 (0.933 : 0.957)
Positive attitude towards health care and medication	25.49 (2.49)	25.68 (2.23)	0.846 (0.810 : 0.875)
Lack of discipline	23.88 (4.69)	23.88 (4.69)	-
Aversion towards medication	15.64 (5.51)	15.46 (5.75)	0.963 (0.953 : 0.970)
Active coping with health problems	18.36 (4.40)	19.06 (3.98)	0.855 (0.821 : 0.883)
* All correlations were significant at the 0.001 level			
